# Effect of Manual Lymph Drainage on Breast Edema After Breast-Conserving Surgery and Radiotherapy: A Preliminary Randomized Controlled Trial

**DOI:** 10.3390/cancers18101510

**Published:** 2026-05-08

**Authors:** Faika Nur Erkol, Nuray Alaca, Nuran Beşe, Cihan Uras

**Affiliations:** 1 Department of General Senology, Senology Research Institute, Acibadem Mehmet Ali Aydinlar University, 34752 Istanbul, Türkiye; erkolfaikanur@gmail.com (F.N.E.); cihan.uras@acibadem.com (C.U.); 2 Department of Physiotherapy and Rehabilitation, Faculty of Health Sciences, Acibadem Mehmet Ali Aydinlar University, 34752 Istanbul, Türkiye; 3 Department of Radiation Oncology, Senology Research Institute, Acibadem Mehmet Ali Aydinlar University, 34752 Istanbul, Türkiye; nuran.bese@acibadem.com; 4 Department of General Surgery, School of Medicine, Acibadem Mehmet Ali Aydinlar University, 34752 Istanbul, Türkiye

**Keywords:** breast cancer, breast-conserving surgery, radiotherapy, breast edema, manual lymphatic drainage, complex decongestive therapy

## Abstract

**Background/Objectives**: To evaluate the effectiveness of manual lymph drainage (MLD) in treating breast edema in patients who have undergone breast-conserving surgery and adjuvant radiotherapy. **Methods**: Twenty-five female participants who underwent breast-conserving surgery and received adjuvant radiotherapy were enrolled in the study. Twelve participants were assigned to the treatment group (education, compression, exercise therapy, and MLD), and 13 to the control group (education, compression, and exercise therapy). The participants were assessed after radiotherapy (baseline) and three months post-treatment. The following variables were evaluated: general body pain, fatigue, breast pain, breast edema (breast edema questionnaire and LENT-SOMA-breast criteria), anxiety, depression, and quality of life. **Results**: In both groups, within-group analyses showed improvements in general body pain, fatigue, breast pain, breast edema, anxiety, depression, and some quality of life subscales (*p* < 0.05). Between-group comparisons revealed additional improvements in breast pain, breast edema, and breast-related criteria in the treatment group compared with the control group (*p* < 0.05). Additionally, the treatment group showed greater improvements in some quality of life subscales (global health status, fatigue, pain, systemic treatment side effects, and breast and arm symptoms) compared with the control group (*p* < 0.05). **Conclusions**: The addition of MLD may provide additional benefits within complex decongestive therapy for breast edema following breast-conserving surgery and radiotherapy. However, these findings should be considered preliminary and interpreted with caution due to the small sample size, lack of objective outcome measures, absence of a no-treatment control group, and lack of blinding of participants and treating clinicians. As both groups received active treatment during a period of expected natural recovery, the independent effect of MLD cannot be fully isolated. Confirmation in future well-designed, blinded randomized controlled trials incorporating objective outcome measures is warranted.

## Simple Summary

Breast-conserving surgery followed by radiotherapy is a common treatment for early-stage breast cancer. Although this approach allows many women to preserve their breast, it can lead to breast edema, a condition characterized by swelling, pain, and changes in breast tissue. Breast edema may negatively affect physical comfort, body image, and quality of life. Despite its frequency, evidence on effective physiotherapy treatments for breast edema remains limited. This study investigated whether adding manual lymph drainage to standard care (education, compression, and exercise therapy) improves outcomes in patients who developed breast edema after breast-conserving surgery and radiotherapy. The results showed that patients who received manual lymph drainage experienced greater improvements in breast pain, breast swelling, and several quality-of-life measures compared with those receiving standard care alone. These findings suggest that manual lymph drainage may be a valuable component of rehabilitation programs for breast cancer survivors experiencing breast edema.

## 1. Introduction

Breast cancer, the most common and lethal cancer among women, has surpassed lung cancer as the leading cause of global cancer incidence, with approximately 2.3 million new cases reported annually [1]. In recent years, the incidence of breast cancer in Turkey has significantly increased, paralleling global trends, with an incidence rate of 47.7 per 100,000 individuals. In Turkey, one in every four women diagnosed with cancer is diagnosed with breast cancer, with 19,211 new cases annually [2]. Breast cancer is treated using a combination of surgical, radiation, and systemic therapies, including chemotherapy and hormonal therapy [3]. Over the past three decades, significant changes have occurred in breast cancer surgery. There has been a shift from radical surgeries to more conservative approaches, with patient-centered decision-making and personalized care becoming paramount [4,5]. Indeed, in comprehensive breast centers, breast-conserving surgery (BCS) rates have reached 75% [6,7,8,9]. Similar trends have been observed in Turkey, with notable variations in surgical practices (rate of BCS = 57% in 2018) [7].

Breast-conserving surgery (BCS) followed by radiotherapy is a well-established, safe, and effective treatment for early-stage breast cancer. While many women experience excellent outcomes, a significant proportion develop breast edema, a condition that is less well understood than arm lymphedema [10,11,12]. Surgery can damage the lymphatic system, impairing lymph fluid drainage not only in the arm but also in the breast. However, radiotherapy is the primary contributor to edema and other tissue reactions. Venous and lymphatic blockages can also exacerbate the condition [13,14]. A systematic review by Verbelen et al. (2014) revealed a wide range of breast edema incidence, from 0% to 90.4%, following BCS and radiotherapy [12]. Another study found that 24.8% of women who underwent BCS experienced breast edema, with the peak incidence occurring six months after radiotherapy [15]. Despite its high prevalence, breast edema following BCS and radiotherapy remains understudied [10,11,12,15].

Common criteria for breast edema in the literature include an increase in breast volume. Additionally, physiological and clinical symptoms of breast edema can include a peau d’orange appearance, a sensation of breast heaviness, skin erythema, breast pain, skin thickening, hyperpigmented skin pores, and a positive pitting sign [10,11,14]. Breast edema is often a painful condition that negatively impacts patients’ personal, social, physical, and professional lives. If left untreated, breast edema can lead to body image issues, fear of cancer recurrence, and psychological distress, significantly reducing quality of life [15,16]. This condition not only causes physical discomfort but also significantly affects women’s body perception and self-esteem. Indeed, many patients experiencing breast edema report significant changes in their clothing choices [12,17,18].

Despite the prevalence of breast edema, its treatment remains inadequately addressed in the literature. Given the increasing rates of breast-conserving surgery (BCS), there is an urgent need for more research on the management of breast lymphedema [10,12,14]. Treatment is often based on knowledge of upper extremity lymphedema management. Lymphedema is typically treated with complex physical therapy consisting of manual lymphatic drainage (MLD), compression therapy, skin care, and exercise [17,19]. However, a recent 2021 systematic review suggested removing MLD from routine treatment protocols due to a lack of research, insufficient evidence of efficacy, high costs, and its time-consuming nature [10]. This has led to questions regarding the routine application of MLD. The only study on this topic, conducted by Jahr et al. in 2008 [19], suggested that deep oscillation in combination with MLD is more effective for breast edema. However, this study only used a visual analog scale for assessment, and the absence of a control group without MLD makes it difficult to draw definitive conclusions about the necessity of MLD. Therefore, the aim of the current study is to determine the effectiveness of MLD in treating breast edema in patients who have undergone breast-conserving surgery and adjuvant radiotherapy. The research question is: Does the addition of MLD to education, skin care, compression, and exercise therapy have any effect on breast edema in patients who have undergone breast-conserving surgery and adjuvant radiotherapy?

## 2. Materials and Methods

### 2.1. Study Design and Participants

This study was designed as a prospective, preliminary, randomized controlled trial and was reported according to the CONSORT statement [20]. After obtaining ethical approval from the Acıbadem University and Acıbadem Healthcare Institutions Medical Research Ethics Committee (ATADEK-2023/18), the study was registered on ClinicalTrials.gov with the identifier NCT05240235. The registration was completed after the initiation of participant recruitment; however, the study protocol had been established prior to enrollment and remained unchanged throughout the study.

Patients aged 18–65 who underwent unilateral breast-conserving surgery, sentinel lymph node biopsy, or axillary lymph node dissection, and adjuvant radiotherapy between November 2023 and June 2024 at the Acıbadem Maslak Hospital Breast Health and Diseases Unit Lymphedema Treatment Center were included if they developed breast edema (Breast Edema Questionnaire ≥ 8.5 points) [11] and met the inclusion criteria. Inclusion was based on the presence and severity of edema rather than the time of onset following radiotherapy. Patients with a history of cancer, decompensated heart failure, untreated congestive heart failure, active infection, uncontrolled hypertension, acute kidney injury, acute deep vein thrombosis, metastasis and/or recurrence during treatment, or those who underwent other surgeries on the breast and chest region were excluded. Additionally, patients with upper extremity lymphedema, as determined by circumferential measurements of both extremities [21], were excluded. Written and verbal informed consent was obtained from all patients in accordance with the Declaration of Helsinki. At the end of the study, since the treatment group had better outcomes, the control group was offered the same treatment free of charge, in accordance with the Declaration of Helsinki.

**Figure 1:**
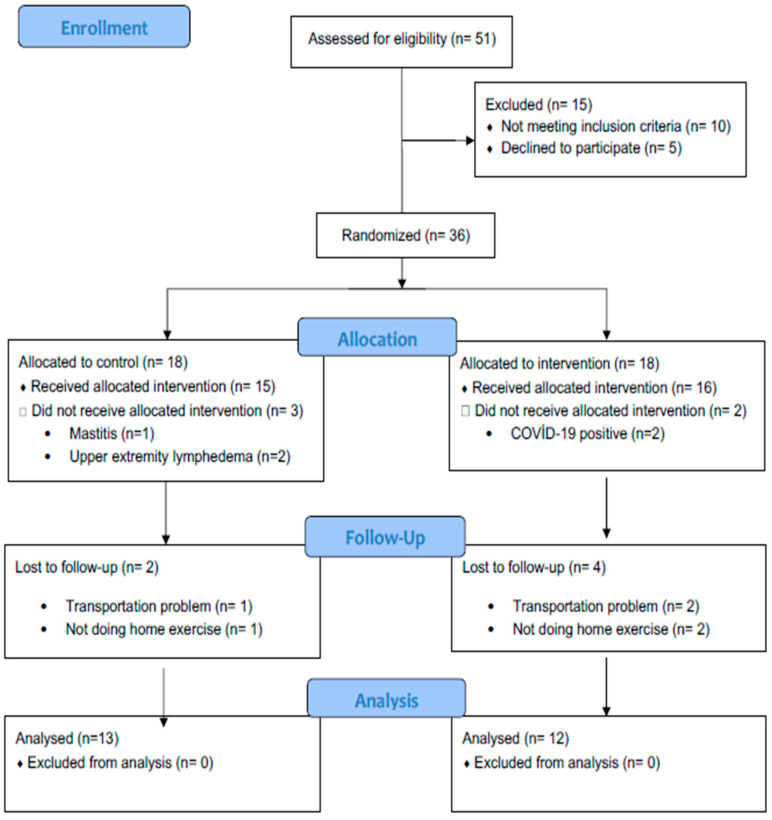
CONSORT flow diagram of patient selection.

To determine the appropriate sample size, G*Power V.3.1.7 (Kiel University, Kiel, Germany) was used. A sample size was calculated to achieve a power of 95%, with a 95% confidence level and a type I error rate of 0.05. Johansson et al. (2020) reported an effect size of 1.4 for the treatment of breast edema in patients with breast edema, and based on this, a total sample size of 24 individuals was determined [22]. Due to the limited number of studies specifically investigating breast edema and the absence of trials involving MLD, this study was selected as the most relevant available source providing sufficient data for effect size estimation. Considering the possibility of patient dropout, a total of 36 patients were planned for enrollment, and the study was completed with 25 participants. In addition, a post hoc power analysis based on the observed effect size indicated a high statistical power (approximately 0.99); however, this should be interpreted with caution due to its dependence on observed effect sizes and should not be considered confirmatory evidence of adequate study power. The flow chart of the study is presented in Figure 1.

### 2.2. Randomization

Before identifying eligible patients, the first author (F.N.E.) and the surgeon (C.U.) informed patients about the study. Patients who met the inclusion criteria were consecutively enrolled and assigned a serial number by the clinical secretary. These serial numbers were entered into a computer-based randomization program (Research Randomizer), which generated the allocation sequence. To ensure allocation concealment, group assignments were managed by the clinical secretary, who was not involved in treatment or outcome assessment, and were communicated to the treating physiotherapist (F.N.E.) only after participant enrollment. According to the generated randomization sequence, participants were assigned to either Group 1 (control) or Group 2 (treatment). The surgeon (C.U.), who performed the LENT-SOMA assessments, was blinded to group allocation. To further minimize bias, statistical analyses were conducted by an independent statistician.

The study groups were as follows:

- Control Group: Group receiving education, compression, and exercise therapy (n = 18)
- Treatment Group: Group receiving education, compression, exercise, and MLD therapy (n = 18)

### 2.3. Interventions

Patients who completed radiotherapy received treatment five times a week for the first two weeks (10 sessions) and once a week for the following 10 weeks (for a total of 20 sessions) by a physiotherapist certified by The Academy of Lymphatic Studies. Patients continued their home exercise programs on the remaining days, and compliance with the home program was monitored daily. Patients who did not adhere to at least 70% of their home program were excluded from the study. The treatments provided to the control and treatment groups are shown in Table 1 [23,24,25].

### 2.4. Assessments

Patients were evaluated at the end of radiotherapy and again three months later, on the last day of the 20th session. The primary outcome measures were a priori defined as the LENT-SOMA scale, the Breast Edema Questionnaire, and the breast cancer-specific quality of life questionnaire (EORTC QLQ-BR23). Secondary outcome measures included pain, fatigue levels, the Hospital Anxiety and Depression Scale, and the generic quality of life questionnaire (EORTC QLQ-C30 version 3.0), and were considered exploratory.

#### 2.4.1. Pain and Fatigue Assessment

General body pain, breast pain severity, and overall fatigue were measured using the Visual Analog Scale (VAS) developed by Price and colleagues. The scale is 10 cm long, with the two ends labeled as 0 = no pain/fatigue and 10 = most severe pain/fatigue. Patients were asked to mark a point on the vertical or horizontal line corresponding to the severity they experienced [26].

#### 2.4.2. LENT-SOMA Breast Criteria

The LENT-SOMA breast criteria is a classification system used to assess the late effects of radiotherapy. LENT-SOMA stands for “Late Effects Normal Tissues-Subjective, Objective, Management, and Analytic” and provides a framework for evaluating late effects in normal tissues following radiotherapy. Breast pain, fibrosis, edema, retraction, skin pigmentation, telangiectasia, ulceration, and arm lymphedema were scored from 1 to 4, with higher scores indicating more severe damage to breast tissue [27]. This assessment was performed by the surgeon (C.U.) before and after treatment. The surgeon was blinded to group allocation during the assessments, and patients were not informed of their group assignment.

#### 2.4.3. Breast Edema Questionnaire

The Breast Edema Questionnaire (BrEQ) was used to assess breast edema before and after treatment. In the first part of the questionnaire, patients rated breast edema symptoms (pain, heaviness, swelling, tense skin, redness, pitting, enlarged skin pores, and hardness) on a scale from 0 to 10. In the second part, in alignment with the International Classification of Functioning, Disability, and Health, patients rated various activity limitations and participation restrictions on a scale from 0 to 10. A cut-off score of 8.5 was used, with scores below 8.5 indicating no breast edema and scores above 8.5 indicating the presence of breast edema [11].

#### 2.4.4. Hospital Anxiety and Depression Scale

The Hospital Anxiety and Depression Scale is used to assess the presence, severity, and progression of anxiety and depression in individuals with physical illnesses and those seeking primary care. The scale consists of 14 items, each rated on a four-point Likert scale. Half of the items assess anxiety symptoms, while the other half assess depressive symptoms. Item scores range from 0 to 3, depending on the severity of the symptoms, and subscale scores are calculated by summing the relevant items. In studies conducted in Turkey, scores of 10–11 or higher on the anxiety subscale and 7–8 or higher on the depression subscale are considered at-risk thresholds [28].

#### 2.4.5. Quality of Life Assessment

The EORTC QLQ-C30 (version 3.0) was developed by Aaronson and colleagues (1993) [29] to assess quality of life. The scale includes three sub-dimensions: global health status, functional scales, and symptom scales with a total of 30 questions. The first 28 questions are scored using a four-point Likert scale, while the 29th and 30th questions are rated on a scale from 1 (very poor) to 7 (excellent). Scores range from 0 to 100, with higher scores on the global health status and functional scales indicating a higher quality of life, while higher scores on the symptom scales reflect a lower quality of life [29,30].

The EORTC-QLQ-BR23 scale consists of 23 questions designed to assess symptoms of the disease and side effects of treatment in breast cancer patients. Scores range from 0 to 100, with higher scores on the functional scale indicating a better quality of life and higher scores on the symptom scale indicating a poorer quality of life [31,32].

#### 2.4.6. Statistical Analysis

Data were analyzed using IBM SPSS Statistics Standard Concurrent User V 26 (IBM Corp., Armonk, NY, USA) statistical software. Descriptive statistics were presented as number (n), percentage (%), and mean ± standard deviation. The normality of numerical variables was assessed using the Shapiro–Wilk test, along with skewness and kurtosis values (acceptable range: −2 to +2). All variables were considered to be normally distributed. Independent samples *t*-test was used to compare numerical descriptive characteristics of patients between groups, and chi-square tests (Pearson chi-square/Fisher exact test) were used to compare categorical descriptive characteristics between groups. Cohen’s d was used to calculate the effect size. Repeated measures ANOVA was used to compare variables between groups over time. In addition, to control for baseline differences in variables that showed statistically significant differences at baseline, analysis of covariance (ANCOVA) was performed using baseline values as covariates. Primary and secondary outcomes were defined a priori. Bonferroni correction was applied to primary comparisons to control for multiple testing, while secondary analyses were considered exploratory and interpreted with caution to avoid an excessive increase in Type II error given the relatively small sample size. A *p*-value < 0.05 was considered statistically significant.

## 3. Results

### 3.1. Participant Flow and Baseline Characteristics

Figure 1 illustrates the flow diagram of the patients. A total of 51 patients were evaluated. Ultimately, the study analyses were completed with 13 patients in the control group and 12 patients in the treatment group. Table 2 summarizes the demographic and clinical characteristics of the patients. No significant differences were found in the baseline characteristics of the groups (*p* > 0.05), except for baseline breast pain and depression scores, which were significantly higher in the treatment group (*p* = 0.002 and *p* = 0.001, respectively).

### 3.2. Pain, Fatigue, Breast Edema, Anxiety, and Depression

Intra-group and intergroup comparisons of pain, fatigue, breast edema, anxiety, and depression parameters are summarized in Table 3. Within-group comparisons revealed a significant improvement in general body pain, breast pain, fatigue, and BrEQ scores after treatment compared to before treatment (*p* < 0.001 for all, Table 3). To account for baseline differences in breast pain, ANCOVA was performed using baseline values as covariates. After adjustment, a significant time × group interaction was observed (F(1,22) = 169.80, *p* < 0.001, η^2^ = 0.885). In the intergroup analysis, no significant differences were found in general body pain [F = 0.498, η^2^ = 0.021, *p* = 0.488], fatigue [F = 1.18, η^2^ = 0.049, *p* = 0.288], anxiety (*p* = 0.166), and depression (*p* = 0.072; ANCOVA-adjusted *p* = 0.499, adjusted for baseline values). However, the treatment group showed significantly lower breast pain [F = 10.1, η^2^ = 0.004, *p* = 0.004]. The BrEQ scores decreased significantly more in the treatment group compared to the control group [F = 44.9, η^2^ = 0.661, *p* < 0.001].

### 3.3. LENT-SOMA Breast Criteria

For the LENT-SOMA criteria, statistical analysis could not be performed for telangiectasia, ulcer, and arm lymphedema, as these were not observed in either group. Intra-group evaluations showed a statistically significant decrease in breast pain (*p* = 0.012 for the control group; *p* < 0.001 for the treatment group) and fibrosis (*p* < 0.001 for all). For edema, a significant reduction was observed only in the treatment group (*p* < 0.001), while the change in the control group was not statistically significant (*p* = 0.273). Similarly, no significant changes were observed in retraction and skin pigmentation in the control group (*p* = 0.165), whereas significant decreases were found in the treatment group (*p* < 0.001 for both parameters) (Table 3). Comparisons between groups indicated a greater reduction in pain [F = 82.3, η^2^ = 0.782, *p* < 0.001], fibrosis [F = 44.9, η^2^ = 0.661, *p* < 0.001], edema [F = 54.1, η^2^ = 0.702, *p* < 0.001], retraction [F = 12.9, η^2^ = 0.360, *p* = 0.002], and pigmentation [F = 92.7, η^2^ = 0.801, *p* < 0.001] in favor of the treatment group (Table 3).

Intra-group comparisons of anxiety and depression scores revealed significant reductions in both groups (*p* < 0.001 for all), but no significant differences were found between the groups [anxiety: F = 2.05, η^2^ = 0.082, *p* = 0.166; depression: F = 0.47, η^2^ = 0.020, *p* = 0.499; Table 3].

### 3.4. Quality of Life Outcomes

Intra-group and intergroup comparisons of quality of life assessments are summarized in Table 4. Statistical analysis could not be performed for hair loss, constipation, and diarrhea symptoms as they were not observed in the patients. For the EORTC QLQ-C30 scores, the global health scale [F = 9.24, η^2^ = 0.287, *p* = 0.006], fatigue [F = 12.50, η^2^ = 0.374, *p* = 0.020], and pain [F = 6.07, η^2^ = 0.209, *p* = 0.022] values were significantly better in the treatment group (Table 4). No significant differences were found between groups for other scores (*p* > 0.05, Table 4). For the EORTC QLQ-BR23 scores, significant differences in favor of the treatment group were found for systemic therapy side effects [F = 30.20, η^2^ = 0.668, *p* < 0.001], breast symptoms [F = 20.20, η^2^ = 0.490, *p* < 0.001], and arm symptoms [F = 28.20, η^2^ = 0.568, *p* < 0.001] (Table 4).

## 4. Discussion

This study aimed to determine whether the addition of manual lymphatic drainage (MLD) to education, skin care, compression, and exercise therapy affects breast edema in patients who underwent breast-conserving surgery and adjuvant radiotherapy. To our knowledge, this is the first randomized controlled study specifically evaluating the effectiveness of MLD in the management of breast edema following breast-conserving surgery and radiotherapy. Although improvements were observed in most parameters, including general body pain, fatigue, breast pain, breast edema, anxiety, depression, and quality-of-life subscales in both groups, patients who received MLD demonstrated significantly greater improvements in breast pain, breast edema symptoms, LENT-SOMA breast criteria, and several quality-of-life domains. These findings suggest that adding MLD may enhance the effectiveness of standard conservative rehabilitation approaches in the management of breast edema.

Importantly, breast edema remains an under-recognized and insufficiently investigated complication compared with upper-extremity lymphedema, despite its considerable impact on patients’ physical comfort, body image, and quality of life. Therefore, the present findings provide important preliminary evidence supporting the potential clinical value of including MLD as a component of complex decongestive therapy in this patient population.

One of the primary treatment approaches for edema following breast cancer surgery is complex decongestive therapy. According to international guidelines from the International Society of Lymphology, complex decongestive therapy—considered the gold standard treatment for lymphedema—has been shown to be effective in reducing swelling and edema [33]. It is widely reported to be an effective method for managing upper-extremity lymphedema following breast cancer [34,35,36]. However, studies specifically investigating the impact of MLD, one of the core components of complex decongestive therapy, remain limited.

A meta-analysis by Liang et al. [37] including patients with upper-extremity lymphedema after breast cancer (n = 1911) reported that MLD did not significantly reduce lymphedema compared with control interventions overall, although a significant benefit was observed in patients younger than 60 years. In the present study, only two patients were older than 60 years (61 and 63 years), while the remaining participants were under 60 years of age. Similarly, Thompson et al. [38] in their meta-analysis (n = 867) emphasized the need for additional experimental studies investigating the effectiveness of MLD and suggested that MLD may not provide additional benefits when combined with complex decongestive therapy in moderate-to-severe lymphedema. A more recent review by Xing et al. (2023) [39] also concluded that larger, well-designed randomized controlled trials are required to further clarify the effectiveness of MLD in the management of breast cancer-related lymphedema.

The number of studies investigating alternative treatments for breast edema remains very limited [10,14]. Various methods such as compression therapy, kinesiology taping, and exercise have been explored [22,40,41,42,43,44]. Johansson et al. (2020) [22] randomized patients to a compression sports bra group and a standard bra group. By the third month following radiotherapy, both groups showed reductions in breast edema and heaviness sensation, with no additional benefit observed from the compression sports bra. In contrast, our study focused specifically on patients with clinically relevant breast edema (BrEQ ≥ 8.5) immediately after radiotherapy and followed them for three months. Differences in patient selection, timing of assessment, and outcome measures may partly explain the discrepancies between the findings.

Gregorowitsch et al. (2020) [40] conducted a pilot study using compression vests in 17 patients and reported that compression vests may represent an acceptable and effective option for painful breast edema. Similarly, in the present study, reductions in breast pain and related symptoms were observed in both groups following three months of compression therapy. However, greater reductions in breast pain, edema symptoms, and breast edema were observed in the group receiving additional MLD. Since correct application of compression bandages can be challenging, compression garments such as vests may represent a more practical alternative in clinical practice. Verbelen et al. (2021) [10] also suggested that the use of compression pads may further enhance treatment outcomes.

Two studies have evaluated the effectiveness of kinesiology taping for breast edema. Finerty et al. (2010) [41] applied kinesiology taping to ten patients for three weeks and reported improvements in breast tissue characteristics, although the absence of a control group limited the interpretation of the results. Ergin et al. (2019) [42] compared MLD alone with MLD combined with kinesiology taping and reported greater reductions in edema with the combined treatment. However, these studies primarily focused on upper-extremity lymphedema rather than breast edema, which may explain differences in findings.

Exercise interventions, including aerobic and resistance training, are consistently reported to have beneficial effects in lymphedema management. A study specifically investigating breast edema demonstrated that a 12-week combined aerobic and resistance exercise program resulted in greater reductions in breast-related symptoms compared with a control group [43]. In our study, improvements were also observed in both groups performing exercise programs, which is consistent with the existing literature.

To date, no randomized controlled trials have investigated the effectiveness of complex decongestive therapy including MLD specifically for breast edema. A review published in 2021 suggested the potential removal of MLD from treatment protocols due to limited research evidence, high cost, and the time-consuming nature of the technique [10]. This recommendation has led to debate regarding the routine use of MLD in clinical practice. The only previous study examining MLD in breast edema was conducted by Jahr et al. (2008) [19], which reported that combining MLD with deep oscillation therapy may improve breast pain but did not significantly reduce breast edema. However, that study lacked a control group, limiting definitive conclusions.

The present study is the first randomized controlled trial specifically evaluating the effect of MLD on breast edema following breast-conserving surgery and radiotherapy. The results indicate that adding MLD to standard treatment may lead to improved outcomes, particularly in reducing breast pain, breast edema symptoms, LENT-SOMA breast criteria scores, and several quality-of-life parameters. These findings suggest that MLD may represent a valuable complementary component in the rehabilitation of women experiencing breast edema. Breast edema developing after breast-conserving surgery and radiotherapy is a frequently overlooked complication that can significantly affect patients’ physical comfort, body image, and quality of life. The present results suggest that adding manual lymph drainage to standard conservative management may provide additional benefits in reducing breast pain, edema-related symptoms, and certain domains of quality of life. These findings indicate that the role of manual lymph drainage within complex decongestive therapy protocols may warrant further consideration in the management of breast edema. It should also be noted that baseline breast pain and depression scores were significantly higher in the treatment group, which may have influenced the magnitude of the observed treatment effects. This difference may also reflect individual variability among participants at baseline. However, this potential confounding effect was controlled for by performing ANCOVA using baseline values as covariates. Furthermore, the significant group × time interaction observed in the repeated measures analysis supports that the observed improvements were related to differences in change over time rather than baseline values alone. Nevertheless, this baseline imbalance should be considered when interpreting the magnitude of the treatment effect.

Several limitations should be acknowledged. These include the relatively small sample size, the lack of blinding of both practitioners and patients, and the absence of objective assessment methods specifically for breast edema. In this study, breast edema was evaluated using patient-reported outcome measures rather than objective methods such as imaging-based or volumetric assessments. Therefore, the findings related to breast edema should be interpreted with caution. Another important limitation is that both groups received active treatment, including education, compression, and exercise therapy. As a result, the absence of a no-treatment control group limits the ability to isolate the specific additional effect of MLD. This raises the possibility that the improvements observed in both groups may partly reflect the effects of standard care or the natural recovery process over time rather than the specific contribution of the intervention. Furthermore, although large effect sizes were observed, particularly in within-group analyses, these values should be interpreted with caution. The relatively small sample size and the magnitude of pre–post changes may have inflated effect size estimates and may not directly reflect the true clinical magnitude of the intervention. Another limitation of this study is the relatively high attrition rate (30.6%), with the final sample decreasing from 36 to 25 participants. This loss to follow-up may be attributed to factors such as COVID-19–related conditions, transportation difficulties, and the vulnerability of patients undergoing cancer treatment. Such attrition may have affected the statistical power and generalizability of the findings. Therefore, the results should be interpreted with caution, and future studies should aim to minimize dropout rates through improved follow-up strategies and patient support. In addition, given the number of statistical comparisons performed across multiple outcome domains, there is a potential risk of Type I error inflation. Although multiple comparison correction was applied to primary outcomes, secondary outcomes were considered exploratory and should be interpreted with caution. The retrospective registration of the trial represents a limitation, as it may affect transparency, although the study protocol had been established prior to participant recruitment. Future large-scale, multicenter randomized controlled trials incorporating objective outcome measures and appropriate control groups are needed to confirm and extend the present findings.

## 5. Conclusions

Improvements were observed across most clinical and patient-reported outcomes in both groups, including general body pain, fatigue, breast pain, breast edema, psychological status, and quality of life. Although the group receiving additional MLD demonstrated greater improvements in several outcomes—particularly breast pain, breast edema-related symptoms, LENT-SOMA breast criteria, and selected quality-of-life domains—these differences should be interpreted with caution, particularly in light of the baseline differences between groups.

Given the relatively small sample size, the preliminary nature of the study, the absence of objective assessment methods for breast edema, and the lack of a no-treatment control group, definitive conclusions regarding the added benefit of MLD cannot be drawn. As both groups received active treatment during a period of expected natural recovery, the independent effect of MLD cannot be fully isolated. Nevertheless, the findings provide preliminary evidence suggesting a potential supportive role of MLD within complex decongestive therapy. Future well-designed randomized controlled trials incorporating larger samples, rigorous blinding procedures, objective outcome measures, and appropriate control conditions are warranted to clarify these effects.

## Figures and Tables

**Table 1:** Treatment program for groups.

Control Group		Treatment Group
Day	Therapy Applications	In Addition to the Therapy Applications Given in the Control Group
***First day*** (Last day of radiotherapy)	Education (1 h) and skin care (with pH 5.5 cream) Upper limb pumping exercises (5 sets of 10 repetitions per day) Wand exercises (3 sets of 10 repetitions per day)	
***Week one*** (5 sessions)	Biopsychosocial education (30 min) ] Bra pad application and usage training where necessary Repeating the skin care training and checking whether skin care is performed Diaphragmatic breathing exercises (5 sets of 5 repetitions per day) Upper limb pumping exercises (5 sets of 10 repetitions per day) Elbow and shoulder range of motion exercises (3 sets of 10 repetitions per day)	MLD (1 h) [23,24,25] = to drain the lymph fluid accumulated in the breast proximally to the lymph nodes in the axillary, supraclavicular and anterior cervical triangle and/or to the lymph nodes on the contralateral side, the following methods are applied according to the patient’s condition. ○ Drainage is started in supine position ○ Stimulation of the cervical lymph nodes with circular movements ○ Stimulation of the supraclavicular lymph nodes with circular movements ○ Breathing exercises for the patient ○ Stimulation of contralateral axillary lymph nodes with circular movements ○ Drainage of the contralateral upper trunk quadrant into the contralateral axillary lymph nodes ○ Anterior Axillo-Axillary watershed opening ○ Drainage of the ipsilateral upper trunk quadrant to the contralateral axillary lymph nodes ○ Stimulation of contralateral axillary lymph nodes ○ Deep pelvic work (diaphragmatic breathing) ○ Stimulation of the ipsilateral inguinal lymph nodes ○ Drainage of the ipsilateral lower trunk quadrant into the ipsilateral inguinal lymph nodes ○ Stimulation of the ipsilateral inguinal lymph nodes ○ Anterior axillo-inguinal watershed opening ○ Drainage of the ipsilateral upper trunk quadrant into the inguinal lymph nodes ○ Stimulation of the ipsilateral inguinal lymph nodes ○ Breathing exercises for the patient and putting the patient in prone position ○ Stimulation of cervical and supraclavicular lymph nodes ○ Stimulation of contralateral axillary lymph nodes ○ Drainage of the contralateral posterior upper trunk quadrant into the contralateral axillary lymph nodes ○ Re-stimulation of contralateral axillary lymph nodes ○ Opening of the posterior axillo-axillary watershed ○ Drainage of the ipsilateral posterior upper trunk quadrant to the contralateral axillary lymph nodes ○ Stimulation of contralateral axillary lymph nodes ○ Putting the patient back in supine position ○ Resolution of soft tissue adhesions around the breast tissue undergoing radiotherapy with manual therapy ○ Breaking of fibrous tissues in the breast tissue by friction massage ○ Drainage of lymph fluid accumulated in the breast tissue to the contralateral lymph nodes ○ Stimulation of bilateral cervical, supraclavicular, axillary, inguinal lymph nodes ○ Breathing exercises for the patient
***Week Two*** (5 sessions)	Biopsychosocial education (30 min) Question and answer session with the patient about education and skin care (half hour) Bra pad application and usage training where necessary Upper limb pumping exercises (5 sets of 10 repetitions per day) Teaching the patient how to relax the whole body before Neck range of motion exercises (1 set of 5 repetitions) Scapular joint range of motion exercises (1 set of 5 repetitions) Shoulder range of motion exercises (1 set of 5 repetitions) Walking exercises (treadmill exercise with 50 per cent of maximum heart rate for 10 min)	
***Between the Third and Twelve Weeks*** (1 day a week under the supervision of a physiotherapist and other days as home exercise)	Checking whether skin care is performed and repeating the training Checking the correct use of the bra pad and repeating the training Relaxation Breathing exercises (1 set of 5 repetitions) Neck range of motion exercises (1 set of 5 repetitions) Scapular joint range of motion exercises (1 set of 5 repetitions) Shoulder range of motion exercises (1 set of 5 repetitions) Elbow range of motion exercises (1 set of 5 repetitions) Pumping exercises (1 set of 5 repetitions) Trunk range of motion exercises (1 set of 5 repetitions) Walking exercises (treadmill exercise with 60–70% of maximum heart rate, increasing from 10 min to 30 min according to the modified borg scale)	MLD (60 min) application once a week as mentioned above Teaching self MLD (20 min) as a home exercise and self-administration by the patient

Control Group: group that received education, compression, and exercise therapy; Treatment Group: group that received education, compression, exercise, and manual lymph drainage therapy.

**Table 2:** Demographic and clinical characteristics of the patients.

Parameters	Control Group	Treatment Group	t or x^2^	*p*
	Mean ± Standard Deviation or n (%)			
**Age**	50.54 ± 9.39	46.42 ± 7.95	t = 1.180	0.250
**Body Mass Index (kg/m^2^)**	23.60 ± 4.15	23.61 ± 3.51	t = −0.738	0.428
**Number of Children (person)**	1.15 ± 0.80	1.67 ± 0.98	t = −1.434	0.165
**Educational status**				*p* = 0.078
*High school*	5 (38.5%)	1 (8.3%)	*χ*^2^ = 3.105	
*University*	8 (61.5%)	11 (91.7%)		
**Menopause before diagnosis**				0.302
*Yes*	7 (53.8%)	4 (33.3%)	*χ*^2^ = 1.066	
*No*	6 (46.2%)	8 (66.7%)		
**Symptom Duration (month)**	2.54 ± 2.40	3.08 ± 2.57	t = −0.547	0.589
**Cancer type**				0.557
*Invasive Ductal Carcinoma*	7 (53.8%)	7 (58.3%)	*χ*^2^ = 2.074	
*Ductal Carcinoma* in Situ	4 (30.8%)	5 (41.7%)		
*Invasive Lobular Carcinoma*	1 (7.7%)	0 (0%)		
*Mucinous Carcinoma*	1 (7.7%)	0 (0%)		
**Tumor stage**				
*Stage 0*	1 (7.7%)	1 (7.7%)	*χ*^2^ = 0.687	0.876
*Stage 1*	9 (69.23%)	8 (66.7%)		
*Stage 2*	2 (15.4%)	1 (7.7%)		
*Stage 3*	1 (7.7%)	2 (15.4%)		
***Tumor Location***				
*Center*	2 (15.4%)	1 (8.3%)	*χ*^2^ = 4.019 ^a^	0.490
*Lower outer quadrant*	1 (7.7%)	3 (25.0%)		
*Upper outer quadrant*	8 (61.5%)	5 (41.7%)		
*Upper inner quadrant*	2 (15.4%)	1 (8.3%)		
*Lower inner quadrant*	0 (0.0%)	2 (16.7%)		
**Size of tumors (cm)**	1.69 ± 0.63	1.75 ± 0.62	t = −0.230	0.820
**Number of Lymph Nodes Removed**	3.31 ± 3.79	4.75 ± 4.33	t = −0.888	0.384
**Positive Lymph Nodes**	0.69 ± 2.21	0.42 ± 0.67	*t =* 0.414	0.683
**Patient Side**				
*Right*	9 (69.2%)	10 (83.3%)	*χ*^2^ = 0.371	0.543
*Left*	4 (30.8%)	2 (16.7%)		
***Number of radiotherapy sessions***	18.80 ± 4.16	20.00 ± 4.66	t = −0.685	0.500
***Total radiotherapy dose (Gy)***	45.20 ± 4.94	43.56 ± 5.87	t = 0.72	0.480
***Boost dose (Gy)***	13.00 ± 8.51	9.38 ± 6.67	t = 1.17	0.250
***Cumulative radiotherapy dose (Gy)***	58.30 ± 9.37	52.81 ± 7.15	t = 1.60	0.120

Control Group: group that received education, compression, and exercise therapy; Treatment Group: group that received education, compression, exercise, and manual lymph drainage therapy; n = number (percentage of frequency); Student’s *t* Test (t); Chi Square Test (*χ*^2^); a. Fisher’s Exact Test.

**Table 3:** Intragroup and Intergroup Comparison of Pain, Fatigue, Breast Edema, Anxiety and Depression Parameters.

Parameters	Groups	Before Treatment	After Treatment	Within-Group Score Change	Effect Size	Intragroup Evaluation (*p*) ^a^	Intergroup Evaluation (*p*) ^b^
		Mean ± Standard Deviation	Mean ± Standard Deviation	[Mean Difference (95% CI, Lower-Upper)]			
**Visual analog scale (0–10 cm)**							
***General body pain***	*Control group*	4.77 ± 0.60	2.23 ± 1.17	2.54 (1.90–3.17)	2.42	**<0.001**	0.488
	*Treatment group*	5.08 ± 1.38	2.67 ± 2.27	2.42 (1.54–3.29)	1.75	**<0.001**	
***Breast pain***	*Control group*	7.69 ± 1.32	5.54 ± 1.27	2.15 (1.74–2.57)	3.13	**<0.001**	**<0.001 ^c^**
	*Treatment group*	9.17 ± 0.72	1.50 ± 0.80	7.67 (7.17–8.16)	9.15	**<0.001**	
***Fatigue***	*Control group*	5.31 ± 1.70	2.00 ± 0.91	3.31 (2.55–4.06)	2.64	**<0.001**	0.288
	*Treatment group*	6.08 ± 1.16	2.25 ± 1.82	3.83 (2.54–5.13)	1.88	**<0.001**	
**Breast Edema Questionnaire**							
	*Control group*	8.85 ± 0.26	6.54 ± 1.77	2.21 (1.20–3.23)	1.32	**<0.001**	**<0.001**
	*Treatment group*	9.15 ± 0.18	2.45 ± 0.78	6.70 (6.14–7.26)	7.57	**<0.001**	
**LENT-SOMA Criteria-Breast**							
**Pain**	*Control group*	2.00 ± 0.82	1.46 ± 0.78	0.54 (0.14–0.94)	0.82	**0.012**	**<0.001**
	*Treatment group*	2.75 ± 0.4	0.08 ± 0.29	2.67 (2.35–2.98)	5.42	**<0.001**	
**Fibrosis**	*Control group*	1.69 ± 0.63	1.08 ± 0.49	0.62 (0.31–0.92)	1.22	**<0.001**	**<0.001**
	*Treatment group*	1.83 ± 0.39	0.00 ± 0.00	1.83 (1.59–2.08)	4.71	**<0.001**	
**Edema**	*Control group*	1.62 ± 0.51	1.38 ± 0.77	0.23 (−0.21–0.67)	0.32	0.273	**<0.001**
	*Treatment group*	1.92 ± 0.29	0.17 ± 0.39	1.75 (1.36–2.15)	2.81	<0.001	
**Retraction**	*Control group*	0.77 ± 0.44	0.62 ± 0.51	0.15 (−0.07–0.38)	0.41	0.165	**0.002**
	*Treatment group*	1.00 ± 0.00	0.25 ± 0.45	0.75 (0.46–1.40)	1.66	**<0.001**	
**Skin Pigmentation**	*Control group*	1.54 ± 0.66	1.38 ± 0.65	0.15 (−0.07–0.38)	0.41	0.165	**<0.001**
	*Treatment group*	1.83 ± 0.39	0.08 ± 0.29	1.75 (1.46–2.04)	3.87	**<0.001**	
**Hospital anxiety and depression scale**							
**Anxiety**	*Control group*	7.92 ± 1.55	3.46 ± 1.20	4.46 (3.88–5.05)	4.61	**<0.001**	0.166
	*Treatment group*	6.75 ± 1.42	3.00 ± 1.41	3.75 (2.81–4.69)	2.53	**<0.001**	
**Depression**	*Control group*	7.54 ± 1.61	3.92 ± 1.44	3.62 (2.78–4.45)	2.61	**<0.001**	0.072 ^c^
	*Treatment group*	5.75 ± 2.05	1.75 ± 1.14	4.00 (3.10–4.90)	2.83	**<0.001**	

CI; confidence interval, Control Group: group that received education, compression, and exercise therapy; Treatment Group: group that received education, compression, exercise, and manual lymph drainage therapy; a. the paired samples *t*-test; b. repeated measurements ANOVA; c. ANCOVA was performed to control for baseline difference; Effect size (Cohen’s d) was interpreted as small (≈0.20), medium (≈0.50), and large (≥0.80). Bold values indicate statistical significance within the group or between groups.

**Table 4:** Intra-group and intergroup comparison of quality of life assessments.

Parameters	Groups	Before Treatment	After Treatment	Within-Group Score Change	Effect Size	Intragroup Evaluation (*p*) ^a^	Intergroup Evaluation (*p*) ^b^
		Mean ± Standard Deviation	Mean ± Standard Deviation	[Mean Difference (95% CI, Lower-Upper)]			
**EORTC QLQ-C30**							
**Global health scale**	*Control group*	31.40 ± 17.10	71.80 ± 12.50	−40.40 (−49.30–31.46)	−2.74	**<0.001**	**0.006**
	*Treatment group*	29.90 ± 14.42	86.80 ± 06.61	−56.90 (−64.70–49.20)	−4.66	**<0.001**	
**Physical functioning**	*Control group*	67.67 ± 11.80	66.95 ± 9.37	0.72 (−3.36–4.80)	0.11	0.708	0.393
	*Treatment group*	66.11 ± 12.86	69.97 ± 8.82	−3.86 (−8.15–0.43)	0.57	0.073	
***Role functioning***	*Control group*	59.70 ± 18.06	100.00 ± 0.00	−40.30 (−51.80–28.80)	−2.23	**<0.001**	0.192
	*Treatment group*	56.90 ± 13.22	100.00 ± 0.00	−43.10 (−51.50–34.70)	−3.20	**<0.001**	
***Emotional functioning***	*Control group*	44.90 ± 11.00	55.10 ± 13.00	−10.30 (−18.80–1.74)	−0.73	**0.022**	0.212
	*Treatment group*	51.40 ± 17.30	70.10 ± 14.80	−18.80 (−30.70–6.78)	−0.99	**0.005**	
***Cognitive functioning***	*Control group*	55.10 ± 10.30	65.40 ± 13.00	−10.30 (−19.90–0.58)	−0.64	**0.040**	0.402
	*Treatment group*	58.30 ± 16.70	75.40 ± 11.20	−16.70 (−30.20–3.12)	−0.78	**0.020**	
***Social functioning***	*Control group*	44.90 ± 14.30	61.50 ± 23.00	−16.70 (−31.50–1.84)	−0.68	**0.031**	0.410
	*Treatment group*	54.20 ± 21.50	79.20 ± 19.00	−25.00 (−41.00–9.04)	0.99	**0.005**	
**Symptom scale**							
**Fatigue symptom**	*Control group*	33.30 ± 18.30	31.50 ± 21.10	1.85 (−4.77–8.47)	0.18	0.551	**0.002**
	*Treatment group*	49.50 ± 21.90	21.20 ± 16.10	28.30 (12.50–44.10)	1.20	**0.003**	
**Nausea/vomiting**	*Control group*	1.28 ± 4.62	0.00 ± 0.00	1.28 (−1.51–4.08)	0.28	0.337	0.955
	*Treatment group*	1.39 ± 4.81	0.00 ± 0.00	1.39 (−1.67–4.45)	0.29	0.339	
**Pain**	*Control group*	44.90 ± 20.80	24.40 ± 27.70	20.50 (12.10–28.90)	1.48	**<0.001**	**0.022**
	*Treatment group*	47.22 ± 19.90	9.72 ± 15.00	37.50 (24.60–50.06)	1.85	**<0.001**	
**Dyspnea**	*Control group*	2.58 ± 9.24	0.00 ± 0.00	2.56 (−3.02–8.15)	0.28	0.337	0.742
	*Treatment group*	4.17 ± 14.40	0.00 ± 0.00	4.17 (−5.00–13.30)	0.29	0.339	
**Insomnia**	*Control group*	35.90 ± 21.40	28.20 ± 18.50	7.69 (−1.14–16.50)	0.53	0.082	0.087
	*Treatment group*	44.30 ± 29.50	16.70 ± 22.50	27.60 (3.92–51.30)	0.74	**0.026**	
**Appetite loss**	*Control group*	12.82 ± 15.40	7.69 ± 14.60	5.13 (−5.26–15.50)	0.30	0.303	0.161
	*Treatment group*	27.80 ± 19.20	11.10 ± 16.40	16.70 (2.39–30.09)	0.74	**0.026**	
**Financial difficulties**	*Control group*	25.64 ± 20.00	12.82 ± 16.90	12.82 (−0.28–25.90)	0.59	0.054	0.325
	*Treatment group*	30.06 ± 17.20	25.00 ± 15.10	5.56 (−2.69–13.80)	0.43	0.166	
**EORTC QLQ-BR23**							
**Body image**	*Control group*	51.90 ± 16.40	64.70 ± 22.30	−12.80 (−23.00–2.62)	−0.76	**0.018**	0.110
	*Treatment group*	25.00 ± 16.70	48.60 ± 27.00	−23.60 (−35.90–11.28)	−1.22	**0.001**	0.178
**Sexual enjoyment**	*Control group*	41.70 ± 16.70	58.30 ± 16.70	−16.70 (−47.30–14.00)	−0.86	0.182	0.407
	*Treatment group*	50.00 ± 17.80	66.70 ± 00.00	−16.70 (−31.60–1.77)	−0.94	**0.033**	
**Future perspective**	*Control group*	20.50 ± 21.70	38.50 ± 26.70	−17.90 (−33.60–02.31)	−0.69	**0.028**	0.419
	*Treatment group*	33.30 ± 01.70	58.30 ± 15.10	−25.00 (−34.60–15.40)	−1.66	**<0.001**	
**Systemic therapy side effects**	*Control group*	48.10 ± 13.10	46.00 ± 15.60	−2.12 (−0.54–4.77)	0.61	0.104	**<0.001**
	*Treatment group*	31.00 ± 5.69	16.40 ± 5.00	15.60 (9.77–19.40)	2.53	**<0.001**	
**Breast symptoms**	*Control group*	80.30 ± 13.10	48.50 ± 22.90	31.80 (14.70–49.00)	1.25	**0.002**	**<0.001**
	*Treatment group*	75.00 ± 12.31	2.78 ± 09.62	72.20 (61.30–83.10)	4.21	**<0.001**	
**Arm symptoms**	*Control group*	33.30 ± 11.88	29.20 ± 05.75	4.17 (−4.34–12.70)	0.41	0.285	**<0.001**
	*Treatment group*	35.35 ± 11.99	2.02 ± 06.70	33.30 (22.80–43.90)	2.12	**<0.001**	

CI: confidence interval; Control Group: group that received education, compression, and exercise therapy; Treatment Group: group that received education, compression, exercise, and manual lymph drainage therapy; a. the paired samples *t*-test; b. repeated measurements ANOVA; mean ± standard deviation; Effect size (Cohen’s d) was interpreted as small (≈0.20), medium (≈0.50), and large (≥0.80). Bold values indicate statistical significance within the group or between groups.

## Data Availability

The datasets generated and analyzed during the current study are available from the corresponding author on reasonable request.

## Abbreviations

The following abbreviations are used in this manuscript:

BCS	Breast-conserving surgery
MLD	Manual lymph drainage
BrEQ	Breast Edema Questionnaire
LENT-SOMA	Late Effects Normal Tissues—Subjective, Objective, Management, and Analytic
EORTC	European Organisation for Research and Treatment of Cancer
QLQ-C30	Quality of Life Questionnaire—Core 30
QLQ-BR23	Quality of Life Questionnaire—Breast Cancer Module 23
HADS	Hospital Anxiety and Depression Scale
VAS	Visual Analog Scale
ANOVA	Analysis of Variance
CI	Confidence interval
